# Quantification of Fusobacterium nucleatum at Depths of Root Dentinal Tubules in the Tooth Using Real-time Polymerase Chain Reaction: An In Vitro Study

**DOI:** 10.7759/cureus.4711

**Published:** 2019-05-21

**Authors:** Arathi Ganesh, Aruna Kumari Veronica, Rupa Ashok, Preethi Varadan, Kandaswamy Deivanayagam

**Affiliations:** 1 Conservative Dentistry and Endodontics, Sri Ramachandra Institute of Higher Education and Research, Chennai, IND; 2 Conservative Dentistry and Endodontics, Madha Dental College, Chennai, IND

**Keywords:** fusobacterium nucleatum, coaggregation, root canal treatment, dentinal tubules, real time polymerase chain reaction

## Abstract

Introduction: Microorganisms have been known to cause pain and infection in the tooth. Fusobacterium nucleatum was always found predominantly in failed root canal treatments.

Objective: The aim of the present study was to quantify Fusobacterium nucleatum at the inner and peripheral half of coronal, middle and apical region of the root by using real-time polymerase chain reaction (qPCR).

Methods: Extracted maxillary incisors were taken. After shaping and cleaning, the root canals were inoculated with Fusobacterium nucleatum. Samples were taken from both the inner and peripheral halves of dentin. The inoculated teeth were maintained in anaerobic jars for two weeks, and the bacterial isolates were changed every third day. The quantification was done using qPCR.

Results: The cycle threshold (Ct) value in all groups showed the presence of Fusobacterium nucleatum.

Conclusion: Fusobacterium nucleatum penetrates to the entire thickness of dentin in the middle and apical region. The coaggregation with other microorganisms could be responsible for the symptomatic endodontic patients.

## Introduction

Microorganisms have been long recognized as the primary cause for the development of periapical lesions and failure of endodontic treatment [1]. Successful endodontic treatment is dependent on the eradication of the infective microflora from the root canal system. Flare-up is defined as the acute exacerbation of asymptomatic pulp or periradicular pathoses after the initiation or continuation of root canal treatment [2]. The reason for failure of root canal treatment is mainly due to procedural errors resulting in lack of control and prevention of the intracanal endodontic infections.

Endodontic failures are usually associated with the persistence of microbial infection in the root canal system and the periradicular area [3]. Fusobacterium nucleatum, one of the main microorganisms is found in root canal infection and periodontal disease [4]. It is one of the most frequently isolated microbes in the root canals of untreated teeth as well as root canal treated teeth with recurrent infection. The virulence of Fusobacterium nucleatum increases when it acts along with other anaerobes [5]. Thus, the aim of the present study is to quantify Fusobacterium nucleatum at both the inner and peripheral halves of coronal, middle and apical regions of the roots using qPCR.

## Materials and methods

In the present study, ten freshly extracted single-rooted maxillary central incisors (extracted for periodontal or prosthodontic reasons) were used. Teeth of uniform length were taken. The roots were decoronated at the level of cementoenamel junction. The roots were treated in an ultrasonic bath containing 3% sodium hypochlorite (NCP Chlorchem, South Africa) for five minutes to remove the debris. The chemical traces used were removed by immersing the roots in an ultrasonic bath containing distilled water for five minutes. The canals were prepared to an apical size of 40 using 2% Kerr Endodontic files (Kerr Corp. Orange, CA, USA). All the roots were sterilized in an autoclave for 20 minutes at 121°C. The roots were 12 ml in length. They were inoculated with ATCC 25586 culture F.nucleatum (Microbiologics Inc., St. Cloud, Minnesota, US; Batch No. 328641) and maintained in anaerobic jars for two weeks. The culture was changed once in every 72 hours. 

Sample preparation

The roots were divided into three portions and samples were taken. The teeth in Group I consisted of samples taken from the coronal third of the tooth. Samples were taken from the middle third and the apical third of the tooth for Group II and Group III, respectively. In each of these groups, samples were taken from the inner and peripheral halves of the root dentin, comprising of Group A and Group B respectively in each group. An autoclaved diamond disk was used to split the tooth vertically into two halves and Gates Glidden drills (Kerr Corp. Orange, CA, USA ) were used to remove dentin from the inner and peripheral region of coronal, middle and apical regions. Group IA-Inner dentinal half in coronal third; Group IB-Peripheral dentinal half in coronal third; Group IIA-Inner dentinal half in middle third; Group IIB-Peripheral dentinal half in middle third; Group IIIA-Inner dentinal half in apical third; Group IIIB-Peripheral dentinal half in apical third.

DNA isolation

The samples were thawed and vigorously vortexed; centrifuged at 8,000x G for five minutes. After the supernatants were removed, the pellets were used for DNA extraction. DNA extraction from dentine samples was done by enzyme extraction method (Bacterial genomic DNA isolation). DNA Isolation protocol used in the present study was adopted with slight modification [6]. 

Specific primer

Primer of 16S rRNA directed specific primers were forward (AGAGTTTGATCCTGGCTCAG) and the reverse primers were (GTCATCGTGCACACAGAATTGCTG).

PCR amplification protocol

The DNA amplification and detection by qPCR is done with specific primer by using 7900HT ABI Real-time PCR Detection System [7]. For each real-time PCR, 20 μl SYBR Green master mix (Thermo Fisher Scientific, Hampton, New Hampshire, United State, US) was used. Total PCR amplification volume for each reaction was placed in each well of a 96-well MicroAmp optical plate (Thermo Fisher Scientific, Waltham, Massachusetts, US) and covered with Optical-Quality sealing tape (Applied Biosystems, Fisher Scientific, Waltham, Massachusetts, USA). The DNA amplification for specific primers was five minutes initial denaturation at 95° C, followed by 40 consecutive cycles at 95° C for 30 seconds, 65° C for 45 seconds, 72° C for 30 seconds, and 72° C for 30 seconds and Ct values were obtained [8]. Ct value represents the amount of target region amplified. Statistical analysis was done by subjecting Ct values to one-way analysis of variance (ANOVA) and T-test.

## Results

The Ct value in Group IA and Group IB were 36.779 and 35.902, respectively, while the Ct value in Group IIA and Group IIB were 36.190 and 36.259, respectively. The Ct value in Group IIIA and Group IIIB were 36.123 and is 36.031, respectively. The Ct value represents the presence of Fusobacterium nucleatum. The statistical analysis is shown in Table 1.

**Table 1:** Statistical analysis using one-way ANOVA and T-Test Group I: Coronal, Group IA-Inner, Group IB-Periphery Group II: Middle, Group IIA-Inner, Group IIB-Periphery Group III- Apical, Group IIIA-Inner, Group IIIB-Periphery

Groups		Mean	Std. Deviation	Sig (2-tailed)
Group I	I A	36.779	0.459	0.004
	I B	35.902	0.700	
Group II	II A	36.190	0.993	0.870
	II B	36.259	0.864	
Group iii	III A	36.123	0.523	0.811
	III B	36.031	1.078	

Bacterial penetration in Group IA was statistically significantly higher than that in Group IB. Equal penetration of the Fusobacterium nucleatum was seen both in the inner and peripheral halves of Group II and Group III. The Fusobacterium nucleatum was seen to penetrate the entire thickness of dentin in the middle and apical regions.

## Discussion

Fusobacterium nucleatum was seen in 48% of the root canals with apical rarefaction [9]. Fusobacterium nucleatum can coaggregate with other microorganisms like Enterococcus faecalis [3]. It can survive and multiply even if as little as 10% of serum is left in between the treatment appointments [10]. Despite being killed during the root canal treatment, the lysed cells present in the dentinal tubule or in the biofilm can act as donors of chromosomal or plasmid DNA. The plasmids or smaller peptides called pheromones can impart drug resistance and virulence to other microbes like Enterococcus faecalis [11], thereby, increasing the pathogenicity of other microorganisms. Studies have shown that the dark pigmented bacteria in pure culture produce mild infection but when it was mixed with Fusobacterium nucleatum, it resulted in abscess and death of animals [12]. Coaggregation of Fusobacterium nucleatum with Enterococcus faecalis suggested a potential role for the combination in endodontic infections [13]. Coaggregation of Fusobacterium nucleatum and other species facilitated the growth of Enterococcus faecalis [14] and increased their number to 27%-56% in nonhealing endodontic cases [15].

Single-rooted central incisors were taken in this study to have uniformity in the sample preparation. The different methods in identification of bacteria into dentinal tubule are culture method, fluorescent microscopy, confocal microscopy, and molecular techniques. The standard culture method used for identification and enumeration of Fusobacterium nucleatum has numerous technical difficulties because of the fastidious nature of the species. Moreover, these methods are extremely laborious, possess lower sensitivity and are time-consuming. Also, the methods usually cannot distinguish the Fusobacterium nucleatum to the subspecies level. Fluorescent microscopy and confocal microscopy can identify bacteria in dentinal tubules but cannot quantify at the depths of dentinal tubule [16]. Molecular approaches have the potential to make the list of endodontic pathogens more accurate. Virulence features can vary among strains of given species and the molecular methods can allow detection of virulent genotypes. Molecular method for microbial typing can allow tracking the origin of root canal bacteria. Application of functional genomics and microarray technologies to the study of endodontic disease includes discrepancy in the host-pathogen interaction in molecular details. It is helpful in identifying target molecules and the pathway for diagnosis and treatment as well as to predict prognosis. Conventional PCR is qualitative but qPCR is quantitative and shows high sensitivity. Hence, qPCR was used in the study.

Results from the current study showed that Fusobacterium nucleatum could penetrate the full thickness of the dentinal tubules in all the groups. A statistically significant difference in the number of bacteria between inner and peripheral halves was seen only in the coronal third of the root canal, with more bacteria seen in the inner half. The reason for this could be the increased thickness of dentin in that area. The present study showed that Fusobacterium nucleatum could penetrate almost the entire depth of dentinal tubule in three weeks.

The current hypothesis on the etiopathogenesis of periapical pathoses implicate both the bacterial and host factors. Fusobacterium nucleatum induces the expression of matrix metalloprotinase (MMP- 13) in host cells infected with bacterium [17] and stimulates the expression of matrix metalloprotinase (MMP-1) as well [18]. In addition, the lipopolysaccharides from Fusobacterium nucleatum triggers the synthesis of interleukin 1α and tumor necrosis factor-α and their release from macrophages [19,20], which in turn might be involved in apical periodontitis-related bone resorption. Further, some of the systemic infections like liver abscess [21] and arthritis [22] were caused by Fusobacterium nucleatum from the dental origin.

Fusobacterium nucleatum is the most frequently associated microorganism in the extraradicular biofilm [6]. Bacterial biofilm was found to develop on root surface outside the apical foramen and associated with apical periodontitis. Studies had suggested that Porphyromonas gingivalis, Tannerella forsythensis and Fusobacterium nucleatum were associated with extraradicular biofilm formation and refractory periodontitis [23]. Biofilms are uniquely suited for horizontal gene transfer [24], and as such might provide an avenue for communication between species of relevance to endodontic infection [25], by plasmid transfer [11]. Therefore, the analysis of the invasiveness of Fusobacterium nucleatum into the dentinal tubules was considered.

The high occurrence of Fusobacterium nucleatum recorded by qPCR might be attributed to its ability to invade the dentinal tubule and adhere to the walls. The present study inferred that Fusobacterium nucleatum had greater invasiveness and could penetrate almost the entire thickness of dentin in apical and middle regions. Further, coaggregation with other microorganisms could be responsible for the symptomatic endodontic cases.

Thus, within the limitations of the study, it is seen that the presence of Fusobacterium nucleatum throughout the entire length of the dentinal tubules, asserts the complexity involved in the eradication of microorganisms during a root canal treatment. Further studies need to be done to find suitable root canal irrigants and irrigants delivery systems to overcome this difficulty.

## Conclusions

Fusobacterium nucleatum penetrates to the entire thickness of dentin in the middle and apical regions. Due to its coaggregation with other microorganisms, it could be responsible for symptomatic endodontic cases. The findings of the present study indicate the need for root canal irrigants and intracanal medicaments that would reach the entire length of the dentinal tubules for the success of the root canal treatment.
